# Congenital Generalized Lipoatrophy (Berardinelli-Seip Syndrome) Type 1: Description of Novel *AGPAT2* Homozygous Variants Showing the Highly Heterogeneous Presentation of the Disease

**DOI:** 10.3389/fendo.2020.00039

**Published:** 2020-02-14

**Authors:** Giovanni Ceccarini, Silvia Magno, Caterina Pelosini, Federica Ferrari, Maria Rita Sessa, Gaia Scabia, Margherita Maffei, Isabelle Jéru, Olivier Lascols, Corinne Vigouroux, Ferruccio Santini

**Affiliations:** ^1^Obesity and Lipodystrophy Center at Endocrinology Unit, University Hospital of Pisa, Pisa, Italy; ^2^Chemistry and Endocrinology Laboratory at University Hospital of Pisa, Pisa, Italy; ^3^Institute of Clinical Physiology, National Research Council, Pisa, Italy; ^4^Sorbonne Université, Inserm UMR_S 938, Centre de Recherche Saint-Antoine, Institut Hospitalo-Universitaire de Cardio-métabolisme et Nutrition (ICAN), Paris, France; ^5^Assistance Publique-Hôpitaux de Paris, Hôpital Saint-Antoine, Laboratoire Commun de Biologie et Génétique Moléculaires, Paris, France; ^6^Assistance Publique-Hôpitaux de Paris, Hôpital Saint-Antoine, Centre National de Référence des Pathologies Rares de l'Insulino-Sécrétion et de l'Insulino-Sensibilité (PRISIS), Service d'Endocrinologie, Diabétologie et Endocrinologie de la Reproduction, Paris, France

**Keywords:** lipodystrophy, Berardinelli-Seip syndrome, BSCL1, *AGPAT2*, leptin

## Abstract

Berardinelli-Seip congenital lipoatrophy (BSCL) is characterized by near total fat atrophy, associated with the progressive development of metabolic complications. BSCL type 1 (BSCL1) is caused by mutations in *AGPAT2*, encoding 1-acylglycerol-3phosphate-O-acyltransferase β (recently renamed lysophosphatidic acid acyltransferase beta), which catalyzes the transformation of lysophosphatidic acid in phosphatidic acid, the precursor of glycerophospholipids and triglycerides. BSCL1 is an autosomal recessive disease due to *AGPAT2* pathogenic variants leading to a depletion of triglycerides inside the adipose organ, and to a defective signaling of key elements involved in proper adipogenesis. We herein investigated the characteristics of two *AGPAT2* variants in Caucasian Italian patients with Berardinelli-Seip congenital lipoatrophy. The first patient exhibited a novel homozygous nonsense c.430 C > T *AGPAT2* mutation (p.Gln144^*^) predicting the synthesis of a truncated enzyme of approximately half of the proper size. The second patient harbored a homozygous *AGPAT2* missense variant (p.Arg159Cys), never described previously in BSCL1 patients: the segregation of the disease with the mutation in the pedigree of the family and the *in silico* analysis are compatible with a causative role of the p.Arg159Cys variant. We remark that BSCL1 can be clinically very heterogeneous at presentation and that the associated complications, occurring in the natural history of the disease, reduce life-expectancy. We point to the necessity for medical treatments capable of reducing the risk of cardiovascular death. In BSCL1 patients, the assessment of cardiovascular disease with conventional diagnostic means maybe particularly challenging.

## Introduction

Congenital generalized lipodystrophy was first described by Waldemar Berardinelli in 1954 (1) and later on further outlined by Martin Seip (2).

Berardinelli-Seip congenital lipoatrophy (BSCL) is characterized by near total fat atrophy since birth, associated with the progressive development of metabolic complications (3). The most common are type 2 diabetes, severe hypertriglyceridemia, acute pancreatitis, hepatic steatosis, and hepatomegaly which are usually detected during infancy and adolescence; other features include, but are not restricted to, muscle pseudo-hypertrophy and acromegaloid appearance, umbilical hernia, polycystic ovary syndrome, cysts in the appendicular bones, cardiopathies, and cardiac rhythm disorders (4, 5). Depending on the underlying molecular cause, mechanical adipose tissue depots of palms, soles, orbits, and under the scalp is preserved or not (6) and other comorbidities can be observed (7).

BSCL is classified in four different subtypes (8); BSCL type 1 is an autosomal recessive disease due to variants (9) of the gene coding for the enzyme 1-acylglycerol-3phosphate-O-acyltransferase β (lysophosphatidic acid acyltransferase beta or *AGPAT2* OMIM# 608594). BSCL type 2 is caused by biallelic mutations in *BSCL2* encoding seipin, a transmembrane protein involved in the functional relationships between endoplasmic reticulum and lipid droplets [OMIM# 269700) (10)]. Rarer forms of BSCL are due to mutations of the CAV1 (OMIM# 612526) and PTRF (OMIM# 613327) genes (11, 12), respectively, encoding caveolin-1 and cavin-1, belonging to the signaling platforms caveolae at the plasma membrane.

*AGPAT2* is a lysophosphatidic acid acyltransferase isoform of 278 amino acids localized in the endoplasmic reticulum and highly expressed in white adipocytes (13). It catalyzes the transformation of lysophosphatidic acid (1,2-diacylglycerol-3phosphate) into phosphatidic acid (glycerol-3-phosphate) which belongs to the glycerophospholipid and triglyceride biosynthetic pathways. *AGPAT2* deficiency leads to a depletion of triglycerides inside the adipose organ and to a defective signaling of key elements such as PI3K/AKT and PPARγ involved in proper adipogenesis (14). *AGPAT2* KO mice develop a phenotype resembling that of BSCL type 1 (BSCL1) in humans characterized by near total loss of white and brown adipose tissue with severe insulin resistance, diabetes, and hepatic steatosis (15).

A very relevant aspect of the disease is the increased food intake caused by reduced circulating levels of the adipose-derived hormone leptin (16), which contributes to the metabolic alterations.

BSCL is a very rare disorder, its prevalence was estimated to be 1–10 case every 10 million (3, 17), although it could reach 3 per 100,000 people in some areas (18). Some of the mutations recur in specific geographic areas, probably due to a “*founder effect*” (19), and their presence is unveiled by consanguineous and endogamic marriages or in small and isolated communities.

Close to 95% of patients affected by BSCL have identified mutations (19). BSCL1 is an autosomal recessive disease and all patients exhibit either homozygous or compound heterozygous *AGPAT2* mutations. Most reported mutations are nonsense or cause altered splicing or frame shifts, which disrupts the protein activity. Only few missense mutations have been so far described.

## Case Report

### Clinical Case N°1

**Figure 1:**
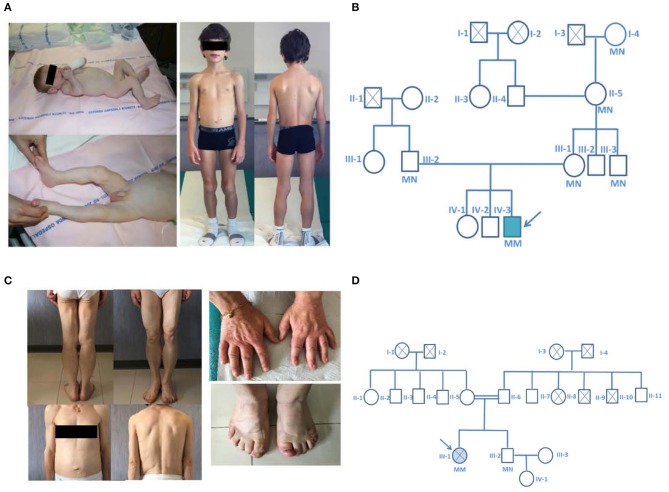
Pictures and family pedigrees of the probands. **(A)** Pictures of Proband 1 at 4 months of age (left) and at close to 9 years of age (right). **(B)** pedigree of the family of the proband affected by a novel nonsense homozygous pathogenic variant (p.Gln144*) in exon 3 of the *AGPAT2* gene. **(C)** Pictures of the proband number 2 and particular of the enlarged hands and feet. **(D)** pedigree of the family of the proband affected by a novel nonsense homozygous variant (Arg159Cys) in exon 3 of the *AGPAT2* gene; parents of the index case were second degree cousins (I-2 and I-4 were first degree cousins). The probands are indicated with the arrow, circles indicate male subjects, squares female subjects. Roman numbers specify the different generations while Arabic numbers identify different subjects. Double horizontal line indicate consanguinity. M, mutant allele; N, normal allele. Filled symbol indicate the presence of the disease, crossed symbols indicate deceased individuals.

A 7 years-old child was referred to our Lipodystrophy Center. The boy was born at term after an uneventful pregnancy. His birth weight was 3.250 Kg. He was the third child of non-consanguineous healthy parents living in a small village in the Northern part of Italy. At the age of 3 months, he was admitted to hospital for vomiting and diarrhea. Physical examination showed a generalized lack of subcutaneous fat, abdominal distension, muscle hypertrophy, low anterior hairline, prominent orbital ridges, large ears, and umbilical hernia (Figure 1). Laboratory tests showed severe hypertriglyceridemia (1565 mg/dl) and increased liver enzymes (AST 543 U/L, ALT 667 U/L), breast feeding was halted and low fat medium chain triglycerides enriched milk was introduced with progressive normalization of the parameters. Liver ultrasound showed diffuse hyperechogenicity consistent with steatosis. BSCL1 syndrome was diagnosed when genetic analysis revealed a novel nonsense homozygous *AGPAT2* pathogenic variant. In this patient a nucleotide change (c.430 C > T) was observed in exon 3 of the *AGPAT2* gene predicting the substitution of the Glutamine residue at position 144 by a stop codon (p.Gln144^*^). This variant was not found in the ExAC nor in the 1,000 genome project databases and has never been described in patients affected by BSCL1. *In silico* analysis confirmed the high pathogenic score of the mutation. The allelic variant in the heterozygous form was found in both asymptomatic parents and in three additional family members of the maternal side (Figure 1).

A low-fat hypocaloric diet was then prescribed. At the time we first evaluated the patient (at 7 years of age) his development and school performance were appropriate for age. On physical examination his height and weight were between the 25th and 50th percentile of the growth charts. He was prepubertal. Lipoatrophy affected the entire body with the exception of the palm of the hands and sole of the feet. He presented muscle hypertrophy, particularly of the calf and thigh muscles (Figure 1). On low fat diet, biochemical analysis showed normal levels of fasting blood glucose, triglycerides, total cholesterol, and liver enzymes. He had low level of high density lipoprotein-cholesterol (HDL-cholesterol 28 mg/dl). As expected from the generalized lack of body fat, serum leptin levels were very low (0.1 ng/ml). Fasting insulin (4.4 mU/L) and glycated hemoglobin levels (33 mmol/mol) were in the normal range. Endocrinological evaluation showed normal thyroid, adrenal and pituitary functions. No pathological findings were demonstrated at cardiologic evaluation (ECG and echocardiography). Abdominal ultrasonography revealed mild steatosis. The hepatic left lobe volume, a standardized surrogate marker of liver volume (20), was enlarged (234 ml). Liver stiffness value assessed by fibroscan was normal (3.7 kPa).

### Clinical Case N°2

The second patient was a 53 years old woman born from consanguineous parents (second-degree cousins, Figure 1) living in a 200 residents village located in the Center of Italy. At 10 years, after development of polyuria and polydipsia she was diagnosed with lipoatrophic diabetes and eruptive xanthomatosis (triglycerides = 718 mg/dl). The patient was advised initially to restrict dietary fats and carbohydrate intake, but over the years she developed uncontrolled hyperglycemia and hyperphagia briefly treated with fenfluramine that exacerbated a bipolar disorder. Later on anti-hypertensive therapy (at age 40) and insulin therapy (at age 43), where administered after she had already developed bilateral proliferative retinopathy and blindness of the left eye because of retinal detachment. At 44 years, she was diagnosed with diabetic and hypertensive nephropathy. She had irregular periods (oligo-menorrhea) throughout her life and no pregnancies. She went into menopause at age of 49.

At the time we first evaluated the patient (53 years of age), physical examination (Figure 1) revealed diffuse reduction of subcutaneous fat, acromegaloid features (marked prognathism and enlarged hands and feet), acanthosis nigricans on the neck and axillae. In addition, she displayed muscle hypertrophy, phlebomegaly and umbilical hernia. No signs of hirsutism were present. The height and the body mass index of the patient were 1.55 m and 22.1 kg/m^2^, respectively.

Biochemical blood tests, under insulin treatment (51 IU/day) and omega three fatty acids, showed high levels of fasting plasma glucose with normal glycated hemoglobin (178 mg/dl; HbA1c 37 mmol/mol, respectively) and frequent hypoglycemic episodes, hypertriglyceridemia (423 mg/dl), and low HDL-cholesterol levels (22 mg/dl). Serum creatinine and proteinuria were 2.14 mg/dl and 1.9 g/24 h, respectively. Endocrinological evaluation showed normal thyroid, parathyroid, and adrenal functions. Plasma leptin and adiponectin were severely reduced (0.4 ng/ml and 0.1 mcg/ml, respectively). Gonadal function showed low levels of gonadotropins with undetectable estradiol (LH 1.1 UI/l; FSH 4.6 UI/l; E2 <20 ng/L) and slightly increased levels of IGF-1 (231 mcg/L) and prolactin (71.4 mcg/l). Pituitary imaging excluded the presence of adenomas or suprasellar masses and hyperprolactinemia was attributed to the chronic treatment with risperidone.

Abdominal ultrasonography revealed mild liver steatosis with an enlarged hepatic left lobe volume (223 ml) and splenomegaly. Body composition assessed by DEXA showed reduced total fat mass (9.2%). Total body X-rays confirmed the presence of multiple bone cysts, confluent, with osteosclerotic appearance, mainly localized in the femoral heads and humerus diaphysis.

Her cardiovascular evaluation showed hypertension, dilated left atrial chamber, left ventricular hypertrophy but normal ventricular volume. Taking into account the high cardiovascular risk of this patient, a stress myocardial perfusion Single-Photon Emission Computed Tomography (SPECT) SPECT was conducted with negative results. The patient had been asymptomatic during the stress test.

Approximately 3 months after discharge from our institution, she experienced chest pain and was admitted to the emergency room and diagnosed with massive myocardial infarction and acute heart failure. Despite transcutaneous revascularization for diffuse coronary stenosis the patient developed progressive cardiac insufficiency and kidney failure. One month after her hospitalization the patient died in the intensive care unit.

In this patient, genetic screening of the *AGPAT2* gene revealed a homozygous gene variant predicting the substitution of the Arginine residue at position 159 with a Cysteine (p.Arg159Cys, nucleotide change c.475C > G).

**Figure 2:**
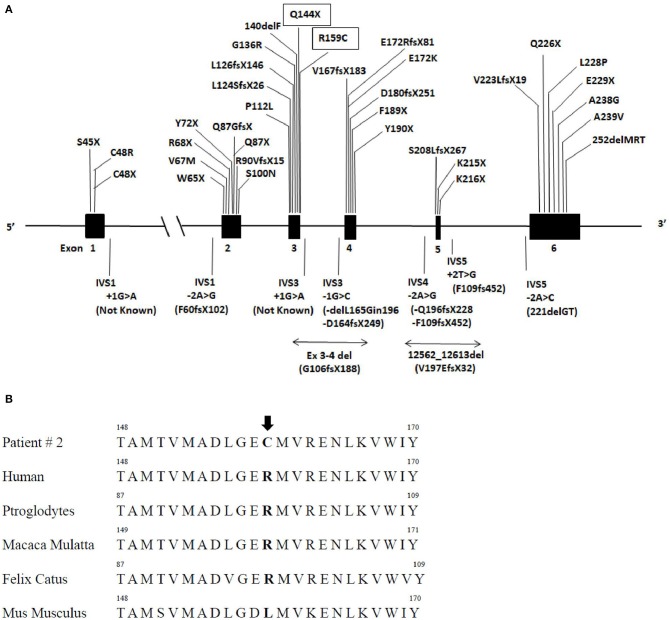
*AGPAT2* gene variants described so far and sequence alignment of patient number 2. **(A)** Schematic gene structure of *AGPAT2* gene, filled boxes indicate exons and in between lines introns: various mutations of *AGPAT2* described so far, novel mutations described in this manuscript are inscribed in rectangular boxes (upper panel). **(B)** Lower panel indicate *AGPAT2* aminoacid sequence alignment in humans and other mammalians. The arrow points at the position of the missense variant at arginine residue 159 (p.Arg159Cys) found in patient number 2 which resides in a highly conserved region.

The missense variant p.Arg159Cys (Figure 2) in exon 3 of the gene was found, among others, in the ExAC database with an allelic frequency of 0.00553 and in the 1,000 Genome Project with an allelic frequency of 0.003 in the general population. *In silico* analysis showed a high pathogenic score (180/215). This aminoacid is significantly conserved (Figure 2) among different species and resides in a highly conserved protein region. Protein stability of the missense variant p.Arg159Cys may be affected as predicted by dedicated software. This variant was found in the heterozygous state in her brother (Figure 1) who had normal triglycerides, glucose tolerance, glycated hemoglobin, hepatic ultrasound, and total body fat assessed by DEXA.

### Biochemistry and Hormones

All determinations were carried out after 12 h fasting. Leptin and adiponectin were measured by CLIA from Mediagnost, Reutlingen, Germany, FT3 (CLIA), FT4 (CLIA), TSH (ICMA) from Ortho Clinical Diagnostic Rochester NY, USA, FSH (ICMA), PRL (ICMA), LH (ICMA), Insulin (ICMA) from Beckman Coulter, Inc. Diagnostics California, USA, and IGF1 (ICMA) from Immunodiagnostic Systems Holdings, UK. Glucose cholesterol, triglycerides, creatinine, AST, ALT, GGT, were determined using automated equipment at the central Laboratory of the University Hospital of Cisanello, Italy.

### Genetic Testing, Mutation Screening

*AGPAT2* variants were identified by next generation sequencing (Patient 1) and/or Sanger sequencing (Patient 2), and all were confirmed by Sanger sequencing in an independent DNA sample. Specific primers were designed to amplify *AGPAT2* exons and splice junctions from genomic DNA, isolated from whole blood. PCR was performed using PCR Master Mix (Promega Corporation, WI 53711-5399) with an annealing temperature of 60°C. After purification with ExoProStar (GE Healthcare UK Limited, UK), the PCR products were directly sequenced using Applied Biosystems 3130 xl sequencer (Thermo Fisher Scientific, MA, USA).

### *In silico* Modeling of the Pathogenicity of *AGPAT2* Variants

*In silico* analysis was conducted using Mutationtaster software [http://mutationtaster.org/ (Last time accessed December 23, 2019)]: each variant is given a score up to the value of 215; a high score is indicative for pathogenic mutations. ExAC and 1,000 Genome Project, dbSNP, Ensembl, Exome Variant server, and in the Clinvar database databases were also searched to determine allelic frequencies in the general population. PROVEAN (Protein Variation Effect Analyzer) is a software tool which predicts whether an amino acid substitution or indel has an impact on the biological function of a protein (http://provean.jcvi.org/index.php, last time accessed December 23, 2019). DUET a server for predicting effects of mutations on protein stability via an integrated computational approach (http://biosig.unimelb.edu.au/duet/stabilityn, last time accessed December 23, 2019) was also used. Prediction from SIFT, Mutation Assessor, CAD and Revel were obtained from the Ensembl genome database.

## Discussion

BSCL1 pathogenic mutations affect triacylglycerol and glycerophospholipid synthesis in adipose tissue and cause lipodystrophy by impairing adipogenesis and depleting the adipocytes of triglycerides (9, 14).

Patients with BSCL1 are either homozygous or compound heterozygous for *AGPAT2* gene mutations that co-segregate with the phenotype of disease in accordance with an autosomal recessive pattern of inheritance. Heterozygotes carriers are asymptomatic although an increased incidence of diabetes is suggested (21).

We have identified 150 cases of patients with a genetically confirmed BSCL1 syndrome reported and 42 different mutations. Most of the mutations cause frame-shifts or alter mRNA splicing leading to the synthesis of a non-functional enzyme while rarer pathogenic variants cause amino acid substitutions (Tables 1, 2). Mutations are spread throughout all six exons and intronic junctions of the gene (Figure 2). Approximately 1/4 of the mutations are nonsense mutations, more frequently present in exon 2 and 3. Insertions and deletions localize usually in exons 3 and 4 while over 80% of mutations altering mRNA splicing localize in intron 4 (p.Gln196fsX228 or p.Phe109fsX452 variants). Fewer patients (15%) present a compound heterozygosity for *AGPAT2* pathogenic variants and each compound mutation, concentrating predominantly in introns or exons 3 or 4, is almost unique. No relationships between *AGPAT2* pathogenic genotypes and the severity of lipodystrophy or specific complications has ever been described.

**Table 1:** Pathogenic variants of *AGPAT2* gene described in the homozygote state.

**Homozygous variants**				
**Exon/intron**	**cDNA**	**Protein**	**Number of cases**	**References**
	**Missense/nonsense**			
Exon 1	c.134 C > A	Ser45X	1	(22)
Exon 1	c.142 C > T	Cys48Arg	2	(23)
Exon 1	c.144 C > A	Cys48X	3	(24)
Exon 2	c.194 G > A	Trp65X	1	(19)
Exon 2	c.199 G > A^*^	Val67Met	1	(25, 26)
Exon 2	c.202 C > T	Arg68X	14	(9, 19, 24, 27, 28)
Exon 2	c.216 C > G	Tyr72X	1	(28)
Exon 2	c.259 C > T	Gln87X	1	(29)
Exon 2 Intron 3	c.299 G > A IVS3-1G > C	Ser100Asn^**^ Asn164fsX249^**^	5	(30–32)^*^
Exon 3	c.335 C > T	Pro112Leu	2	(33, 34)
Exon 3	c.430 C > T	Gln144X	1	Current study
Exon 3	c.475 C > T	Arg159Cys	1	Current study
Exon 4	c.514 G > A	Glu172Lys	5	(19, 22, 35, 36)
Exon 5	c.643A > T^***^	Lys216X	5	(19, 24, 35, 37)^****^
Exon 6	c.676 C > T	Gln226X	1	(19)
Exon 6	c.685 G > T	Glu229X	5	(22, 24)
	**Deletion**			
Exon 2	268delC	Arg90ValfsX15	1	(24)
Exon 3	c.369_372deLGCTC	Leu124SerfsX26	1	(38)
Exon 3-4	317-588del (Ex 3-4del)	Gly106fsX188	24	(9, 19, 37, 39, 40)
Exon 6	755TGAGGACCA del	252delMetArgThr	1	(30)
Intron 4-5	12562_12613 del	Val197GlufsX32	1	(41)
Intron 5	IVS5-2A>C	221delGlyThr	2	(9, 24)
	**Insertion**			
Exon 2	258_259insGGCTG	Gln87GlyfsX	1	(42)
Exon 3	377insT	Leu126fsX146	1	(30)
	**Deletion/insertion**			
Exon 6	667_705delinsCTGCG	Val223LeufsX19	2	(24)
	**Splice-site**			
Intron 2	IVS2+1G > T	–	2	(24)
Intron 3	IVS3-1G > C	delLeu165-Gin196	2	(19, 35)
Intron 3	IVS3-1G > C	Asn164fsX249	3	(32)
Intron 4	IVS4-2A > G	Gln196fsX228	23	(9, 28, 30, 35, 37)
Intron 4	IVS4-2A > G	Phe109fsX452	16	(19)
Intron 5	IVS5+2T > G	Phe109fsX452	2	(19)

* *Originally described as c.119 G > A*.

** *Double homozygosity*.

*** *Originally described as 712 A > T*.

**** *Originally described as 645 A > T*.

**Table 2:** Pathogenic variants of *AGPAT2* gene in the compound heterozygous state.

**Compound heterozygote variants**				
**Exon**	**cDNA**	**Protein**	**Number of cases**	**References**
Exon 2 Intron 4	202C > T IVS4-2A > G	Arg68X Gln196fsX228	2	(43)
Exon3 Intron 4	c.369_372delGCTC IVS4-2A > G	Leu124SerfsX26 –	1	(38)
Exon 3 Exon 4	406G > A 504delGA	Gly136Arg Val167fsX183	1	(9)
Exon 3 Intron 4	418delTTC IVS4-2A > G	140delPhe Gln196fsX228	1	(9)
Exon 3 Intron 4	377insT IVS4-2A > G	Leu126fsX146 Gln196fsX228	2	(9)
Exon 3 Intron 4	355 C > T IVS4-2A > G	Pro112Leu Gln196fsX228	1	(43)
Exon 4 Exon 5	c.513del C c.622_626TCCTC	Glu172ArgfsX81 Ser208LeufsX267	1	(44)
Exon 4 Intron 1	538Gdel IVS1+1G > A	Asp180fsX251 –	1	(30)
Exon 4 Intron 3	c.636 C > A IVS3-1G > C	Phe189X Asn164fsX249	1	(37)
Exon 4 Intron 4	570 C > A IVS4-2A > G	Tyr190X Gln196fsX228	2	(30)
Exon 6 Intron 4	683T > C IVS4-2A > G	Leu228Pro Gln196fsX228	2	(9)
Exon 6 –	c. 712 C > G –	Ala238Gly –	1	(19)
Exon 6 Exon 6-3'UTR	716 C > T c.916 C > G	Ala239Val –	1	(9)
Intron 1 –	IVS1-2A > G –	Phe60fsX102 –	1	(19)
Intron 4 Intron 3	IVS4-2A > G IVS3+1G > A	Gln196fsX228 –	1	(30)
Intron 4 –	IVS4-2A > G –	Phe109fsX452 –	2	(19)

Several are the metabolic complications related to *AGPAT2* functional loss, insulin resistance is a hallmark of the disease and usually progresses during childhood (4) leading to diabetes mellitus after adolescence.

In the functional absence of *AGPAT2*, liver activity of the isoform 1 of the enzyme (*AGPAT1*) is significantly upregulated leading to overproduction of diacylglycerol, used for triacylglycerol synthesis (15). This could be an additional mechanism responsible for the development of severe liver steatosis and hepatomegaly besides the ectopic accumulation of triglycerides of alimentary origin.

Patients with BSCL1 display specific skeletal abnormalities, in particular lytic lesions at the extremities of appendicular bones associated with a diffuse serous transformation of the bone marrow (35), which can help clinicians with an early diagnosis in pauci-symptomatic patients.

Many patients with generalized lipodystrophy show evidence of cardiac hypertrophy, as measured by echocardiographic parameters. Lupsa et al. (45) have documented that out of 19 patients affected by BSCL1, 10 had some evidence, in some cases severe, of left ventricular hypertrophy. ECG abnormalities were seen in 53% of patients.

At variance with the numerous cardiovascular risk factors displayed by patients with congenital generalized lipodystrophy (46), myocardial infarction and sudden death were not reported to be the leading cause of decease (47).

The two cases presented illustrate well the clinical heterogeneity of BSCL1. In the younger patient, born from non-consanguineous parents, the metabolic complications, at the age of 8 years, were mild and controlled by hypocaloric diet and physical activity. The older woman, who was diagnosed during puberty, showed the multiple and severe metabolic derangements typically occurring in the natural history of the disease. Regarding the cardiac health, ECGs in both our patients were normal but echocardiography revealed dilated left atrial chamber and left ventricular hypertrophy in the adult patient, who also had hypertension. Taking into account the numerous and long standing cardiovascular risk factors of this 53 years old woman, we performed one of the most accurate test available for diagnosing coronary artery disease, which, however, failed to foresee the underlining ischemic disease. The etiology of the frequent hypertrophic cardiomyopathy in lipodystrophy remains unclear; in part, it could be related to the underlying hypertension, but also to the increased growth stimulatory action of excess insulin (48). A third potential mechanism of cardiac disease is related to the increased fatty acid influx or ectopic deposition of fat in the myocardium. “Lipotoxic cardiomyopathy” in lipodystrophy has not proved yet but is a very plausible explanation since a significant number of patients develop cardiac hypertrophy and left ventricular dysfunction in the absence of hypertension (45). Lipotoxic cardiomyopathy, together with the vascular complications of diabetes, hypertension and dyslipidemia, favor cardiovascular morbidity and mortality (46). Retrospectively, the adoption of an insulin treatment favoring hypoglycemic episodes rather than insulin sensitizers and drugs with a protective cardiovascular profile may have been further detrimental in this case. Furthermore, the role for implementation of a protective cardiac treatment (ace-inhibitors, anti-aldosterone drugs, anti-aggregants, PCSK9 inhibitors) should be assessed in adult patients with BSCL.

Another consequence of chronically decreased leptin levels is the reduction of GnRH pulsatile activity which causes oligo-menorrhea (4). Indeed, our patient presented with a clinical history of oligo-menorrhea and showed almost undetectable gonadotropin levels, probably determined by the combination of reduced leptin levels and iatrogenic hyperprolactinemia. Notably, ~10% of BSCL1 patients, display cognitive impairment (49), but our patients did not.

Described individuals with mutations in the *AGPAT2* gene mainly originated from America or Northern Europe and occasionally from sub-Saharan Africa or Middle Eastern Countries (50). As for Caucasian Italian population the two herein described subjects add up to one other single case previously reported (33).

The first mutation (p.Gln144^*^) is not present in databases collecting variants identified in the general population.

In this case it is straightforward to predict that the mutation affects the protein activity via the synthesis of a truncated enzyme approximately half (143 amino acids) of the proper size. Missense mutations located close (p.Gly136Arg) (9) or nonsense mutations more distal to the C-terminal part of the protein have been previously reported as pathogenic (e.g., p.Phe189X, p.Tyr190X, p.Lys216X, p.Gln226X) (19, 30, 37).

The substitution of the Arginine residue at position 159 with a Cysteine (*AGPAT2* p.Arg159Cys) was present in the homozygous state in Proband 2 and found in the heterozygous state in the unaffected brother. This variation alters a conserved amino acid site in a highly conserved region among mammalian species and its *in silico* score was predictive of disruption of protein activity. Furthermore, the homozygous variant segregated with the disease in the family making its pathogenic role possible.

We speculate that the substitution of hydrophilic charged Arginine with a polar, non-charged Cysteine, capable of forming disulfide bonds may disrupt the folding or activity of the enzyme. Results from dedicated software tools are not fully concordant in this regard: SIFT, Mutation Assessor, CAD, and PROVEAN predict in fact the aminoacid change to be neutral whereas Revel consider this substitution pathogenic, in line with the reduced stability of the R159C variant predicted by DUET, an integrated computational approach that takes into account the three dimensional structure of the protein.

Databases collecting variants existent in the general population report the heterozygous p.Arg159Cys to be present at a frequency around 0.3% which is higher than expected from the disease prevalence. The presence of few homozygous carriers (three subjects in ExaC and nine subjects in gnomAD) is reported by these databases but no information regarding the clinical history of the homozygous subjects can be retrieved. This evidence is difficult to reconcile with our findings. We therefore cannot rule out different pathogenic mechanisms. Pathogenic *AGPAT2* missense mutations are thought to reduce protein expression (23) and possibly enzymatic activity (27). Testing *in vitro* the effects of the described mutation may give us further clues, but the assessment of the pathogenic potential of *AGPAT2* variants has been proven challenging and has been rarely performed (23, 27).

Berardinelli Seip syndrome is frequently unveiled by consanguineous marriages or in small and relatively isolated communities (50). This was indeed the case for both patients. The first one was born from apparently unrelated parents living in a small village of few thousands inhabitants. The occurrence of a unique variant of *AGPAT2* was found in members on both sides of the family, suggesting a founder effect from a common ancestor. The second patient was born from second-degree cousins.

In conclusion we describe two patients with BSCL type 1 lipodystrophy harboring novel homozygous variants. The first one presented with a null variant (p.Gln144^*^) occurring in exon 3 of *AGPAT2* gene. The second patient presented with a homozygous *AGPAT2* missense variant (p.Arg159Cys), never described previously in BSCL1 patients, raising the question on its pathogenicity. The segregation of the disease with the genotype in the family and the *in silico* analysis are compatible with a causative role of the p.Arg159Cys variant.

We remark that BSCL1 can be clinically very heterogeneous at presentation and that the associated complications, occurring in the natural history of the disease, may reduce life-expectancy. We point to the necessity for medical treatments capable of reducing the risk of cardiovascular death. In BSCL1 patients, the assessment of cardiovascular disease with conventional diagnostic means maybe particularly challenging. Future studies will be essential to investigate if specific cardiovascular diagnostic approach is recommended in adult BSCL patients.

## Data Availability Statement

All datasets generated for this study are included in the article.

## Ethics Statement

Written informed consent to participate in this study was provided by the participants' legal guardian/next of kin. Written informed consent was obtained from the individual(s), and minor(s)' legal guardian/next of kin, for the publication of any potentially identifiable images or data included in this article.

## Author Contributions

GC, SM, FF, and FS carried out the characterization of the patients. CP, MS, GS, MM, and OL performed the genetic studies of *AGPAT2* gene. GC, SM, and FS wrote the manuscript. IJ and CV reviewed and edited the manuscript. All authors read and approved the final version of the manuscript.

### Conflict of Interest

The authors declare that the research was conducted in the absence of any commercial or financial relationships that could be construed as a potential conflict of interest. The reviewer GA declared a past co-authorship with the authors GC and FS to the handling editor.

## Abbreviations

BSCL: Berardinelli-Seip congenital lipoatrophy
*AGPAT2*: 1-acylglycerol-3phosphate-O-acyltransferase β
PI3K: phosphoinositide 3-kinase
AKT: serine/threonine-protein kinases
OMIM: online mendelian inheritance in man
PPARγ: peroxisome proliferator-activated receptor gamma
CAV1: caveolin-1
PTRF: polymerase I and transcript release factor
KO: knockout mouse
AST: aspartate aminotransferase
ALT: alanine aminotransferase
ExAC: exome aggregation consortium
HDL: high density lipoprotein-cholesterol
ECG: electrocardiogram
HbA1c: glycated hemoglobin
LH: luteinizing hormone
FSH: follicle-stimulating hormone
E2: estradiol
IGF-1: Insulin-like growth factor-1
DEXA: dual-energy x-ray absorptiometry
FT3: free tri-iodothyronine
CLIA: chemiluminescence immunoassay
FT4: free thyroxine
TSH: thyroid stimulating hormone
ICMA: chemiluminescent immunochemiluminometric assay
PRL: prolactin
GGT: gamma-glutamyl transferase
PCR: polymerase chain reaction
dbSNP: Single Nucleotide Polymorphism Database.

## References

[1] Berardinelli W. An undiagnosed endocrinometabolic syndrome: report of 2 cases. J Clin Endocrinol Metab. (1954) 14:193–204. 10.1210/jcem-14-2-193 13130666

[2] Seip M. Lipodystrophy and gigantism with associated endocrine manifestations. A new diencephalic syndrome? Acta Paediatr Scand. (1959) 48:555–74. 10.1111/j.1651-2227.1959.tb17558.x 14444642

[3] Garg A. Acquired and inherited lipodystrophies. N Engl J Med. (2004) 350:1220–34. 10.1056/NEJMra025261 15028826

[4] Garg A. Lipodystrophies: genetic and acquired body fat disorders. J Clin Endocrinol Metab. (2011) 96:3313–25. 10.1210/jc.2011-1159 21865368 PMC7673254

[5] Capeau J Magré J Caron-Debarle M Lagathu C Antoine B Béréziat V. Human lipodystrophies: genetic and acquired diseases of adipose tissue. Endocr Dev. (2010) 19:1–20. 10.1159/000316893 20551664 PMC3892722

[6] Simha V Garg A Phenotypic heterogeneity in body fat distribution in patients with congenital generalized lipodystrophy caused by mutations in the AGPAT2 or seipin genes. J Clin Endocrinol Metab. (2003) 88:5433–7. 10.1210/jc.2003-030835 14602785

[7] Akinci B Meral R Oral EA. Phenotypic and genetic characteristics of lipodystrophy: pathophysiology, metabolic abnormalities, and comorbidities. Curr Diab Rep. (2018) 18:143. 10.1007/s11892-018-1099-9 30406415

[8] Araújo-Vilar D Santini F. Diagnosis and treatment of lipodystrophy: a step-by-step approach. J Endocrinol Invest. (2018) 42:61–73. 10.1530/endoabs.56.S20.3 29704234 PMC6304182

[9] Agarwal AK Arioglu E De Almeida S Akkoc N Taylor SI Bowcock AM. AGPAT2 is mutated in congenital generalized lipodystrophy linked to chromosome 9q34. Nat Genet. (2002) 31:21–3. 10.1038/ng880 11967537

[10] Magré J Delépine M Khallouf E Gedde-Dahl T Jr Van Maldergem L Sobel E. Identification of the gene altered in Berardinelli-Seip congenital lipodystrophy on chromosome 11q13. Nat Genet. (2001) 28:365–70. 10.1038/ng585 11479539

[11] Kim CA Delépine M Boutet E El Mourabit H Le Lay S Meier M. Association of a homozygous nonsense caveolin-1 mutation with Berardinelli-Seip congenital lipodystrophy. J Clin Endocrinol Metab. (2008) 93:1129–34. 10.1210/jc.2007-1328 18211975

[12] Hayashi YK Matsuda C Ogawa M Goto K Tominaga K Mitsuhashi S. Human PTRF mutations cause secondary deficiency of caveolins resulting in muscular dystrophy with generalized lipodystrophy. J Clin Invest. (2009) 119:2623–33. 10.1172/JCI38660 19726876 PMC2735915

[13] Shindou H Hishikawa D Harayama T Yuki K Shimizu T. Recent progress on acyl CoA: lysophospholipid acyltransferase research. J Lipid Res. (2009) 50:S46–51. 10.1194/jlr.R800035-JLR200 18931347 PMC2674719

[14] Subauste AR Das AK Li X Elliot B Evans C El Azzouny M. Alterations in lipid signaling underlie lipodystrophy secondary to AGPAT2 mutations. Diabetes. (2012) 61:2922–31. 10.2337/db12-0004 22872237 PMC3478532

[15] Cortés VA Curtis DE Sukumaran S Shao X Parameswara V Rashid S. Molecular mechanisms of hepatic steatosis and insulin resistance in the AGPAT2 deficient mouse model of congenital generalized lipodystrophy. Cell Metab. (2009) 9:165–76. 10.1016/j.cmet.2009.01.002 19187773 PMC2673980

[16] Triantafyllou GA Paschou SA Mantzoros CS. Leptin and hormones: energy homeostasis. Endocrinol Metab Clin North Am. (2016) 45:633–45. 10.1016/j.ecl.2016.04.012 27519135

[17] Chiquette E Oral EA Garg A Araújo-Vilar D Dhankhar P. Estimating the prevalence of generalized and partial lipodystrophy: findings and challenges. Diabetes Metab Syndr Obes. (2017) 10:375–83. 10.2147/DMSO.S130810 29066925 PMC5604558

[18] De Azevedo Medeiros LB Cândido Dantas VK Craveiro Sarmento AS Agnez-Lima LF Meireles AL Xavier Nobre TT. High prevalence of Berardinelli-Seip Congenital Lipodystrophy in Rio Grande do Norte State, Northeast Brazil. Diabetol Metab Syndr. (2017) 9:80. 10.1186/s13098-017-0280-7 29046728 PMC5640922

[19] Magré J Delépine M Van Maldergem L Robert JJ Maassen JA Meier M. Prevalence of mutations in AGPAT2 among human lipodystrophies. Diabetes. (2003) 52:1573–8. 10.2337/diabetes.52.6.1573 12765973

[20] Giannetti M Piaggi P Ceccarini G Mazzeo S Querci G Fierabracci P. Hepatic left lobe volume is a sensitive index of metabolic improvement in obese women after gastric banding. Int J Obes. (2012) 36:336–41. 10.1038/ijo.2011.243 22143620

[21] Agarwal AK Garg A. Genetic disorders of adipose tissue development, differentiation, and death. Annu Rev Genomics Hum Genet. (2006) 7:175–99. 10.1146/annurev.genom.7.080505.115715 16722806

[22] Haghighi A Razzaghy-Azar M Talea A Sadeghian M Ellard S Haghighi A. Identification of a novel nonsense mutation and a missense substitution in the AGPAT2 gene causing congenital generalized lipodystrophy type 1. Eur J Med Genet. (2012) 55:620–24. 10.1016/j.ejmg.2012.07.011 22902344 PMC3471069

[23] Ramanathan N Ahmed M Raffan E Stewart CL O'Rahilly S Semple RK Identification and characterisation of a novel pathogenic mutation in the human lipodystrophy gene AGPAT2: C48R: a novel mutation in A*GPAT2*. JIMD Rep. (2013) 9:73–80. 10.1007/978-3-642-35518-9_196 23430550 PMC3565662

[24] Akinci B Onay H Demir T Ozen S Kayserili H Akinci G. Natural history of congenital generalized lipodystrophy: a nationwide study from Turkey. J Clin Endocrinol Metab. (2016) 101:2759–67. 10.1210/jc.2016-1005 27144933 PMC7958923

[25] Poovazhagi V Shanthi S Jahnavi S Radha V Mohan V Berardinelli Seip congenital lipodystrophy presenting with neonatal diabetes mellitus due to a mutation in the AGPAT2 gene. Int J Diabetes Dev C. (2013) 33:66–8. 10.1007/s13410-012-0099-6

[26] Jahnavi S Poovazhagi V Mohan V Bodhini D Raghupathy P Amutha A. Clinical and molecular characterization of neonatal diabetes and monogenic syndromic diabetes in Asian Indian children. Clin Genet. (2013) 83:439–45. 10.1111/j.1399-0004.2012.01939.x 22831748

[27] Haque W Garg A Agarwal AK. Enzymatic activity of naturally occurring 1-acylglycerol-3-phosphate-O-acyltransferase 2 mutants associated with congenital generalized lipodystrophy. Biochem Biophys Res Commun. (2005) 327:446–53. 10.1016/j.bbrc.2004.12.024 15629135

[28] Haghighi A Kavehmanesh Z Haghighi A Salehzadeh F Santos-Simarro F Van Maldergem L. Congenital generalized lipodystrophy: identification of novel variants and expansion of clinical spectrum. Clin Genet. (2016) 89:434–41. 10.1111/cge.12623 26072926 PMC7672659

[29] Debray FG Baguette C Colinet S Van Maldergem L Verellen-Dumouin C. Early infantile cardiomyopathy and liver disease: a multisystemic disorder caused by congenital lipodystrophy. Mol Genet Metab. (2013) 109:227–9. 10.1016/j.ymgme.2013.04.011 23647707

[30] Agarwal AK Simha V Oral EA Moran SA Gorden P O'Rahilly S. Phenotypic and genetic heterogeneity in congenital generalized lipodystrophy. J Clin Endocrinol Metab. (2003) 88:4840–7. 10.1210/jc.2003-030855 14557463

[31] Cortés VA Smalley SV Goldenberg D Lagos CF Hodgson MI Santos JL. Divergent metabolic phenotype between two sisters with congenital generalized lipodystrophy due to double AGPAT2 homozygous mutations. a clinical, genetic and *in silico* study. PLoS ONE. (2014) 9:e87173. 10.1371/journal.pone.0087173 24498038 PMC3909042

[32] Miranda DM Wajchenberg BL Calsolari MR Aguiar MJ Silva JM Ribeiro MG. Novel mutations of the BSCL2 and AGPAT2 genes in 10 families with Berardinelli-Seip congenital generalized lipodystrophy syndrome. Clin Endocrinol. (2009) 71:512–7. 10.1111/j.1365-2265.2009.03532.x 19226263

[33] Pelosini C Martinelli S Bagattini B Pucci E Fierabracci P Scartabelli G. Description of an AGPAT2 pathologic allelic variant in a 54-year-old Caucasian woman with Berardinelli-Seip syndrome. Acta Diabetol. (2011) 48:243–6. 10.1007/s00592-011-0308-7 21744063

[34] Ben Turkia H Tebib N Azzouz H Abdelmoula MS Ben Chehida A Hubert P. Congenital generalized lipodystrophy: a case report with neurological involvement. Arch Pediatr. (2009) 16:27–31. 10.1016/j.arcped.2008.10.005 19026526

[35] Teboul-Coré S Rey-Jouvin C Miquel A Vatier C Capeau J Robert JJ. Bone imaging findings in genetic and acquired lipodystrophic syndromes: an imaging study of 24 cases. Skeletal Radiol. (2016) 45:1495–506. 10.1007/s00256-016-2457-9 27631079

[36] Rostami P Nakhaeimoghadam M Bijani FM Sotoudeh A Rabbani A Hilbert P. AGPAT2 gene mutation in a child with Berardinelli-Seip congenital lipodystrophy syndrome. Ann Endocrinol. (2013) 74:59–61. 10.1016/j.ando.2012.11.008 23337016

[37] Fu M Kazlauskaite R Baracho Mde F Santos MG Brandão-Neto J Villares S. Mutations in Gng3lg and AGPAT2 in Berardinelli-Seip congenital lipodystrophy and Brunzell syndrome: phenotype variability suggests important modifier effects. J Clin Endocrinol Metab. (2004) 89:2916–22. 10.1210/jc.2003-030485 15181077 PMC3390418

[38] Ponte CMM Fernandes VO Gurgel MHC Vasconcelos ITGF Karbage LBAS Liberato CBR. Early commitment of cardiovascular autonomic modulation in Brazilian patients with congenital generalized lipodystrophy. BMC Cardiovasc Disord. (2018) 18:6. 10.1186/s12872-017-0738-4 29329523 PMC5767058

[39] Gomes KB Fernandes AP Ferreira AC Pardini H Garg A Magré J. Mutations in the seipin and AGPAT2 genes clustering in consanguineous families with Berardinelli-Seip congenital lipodystrophy from two separate geographical regions of Brazil. J Clin Endocrinol Metab. (2004) 89:357–61. 10.1210/jc.2003-030415 14715872

[40] Barra CB Savoldelli RD Manna TD Kim CA Magre J Porta G. Genetic and metabolic description of five patients with Berardinelli-Seip syndrome. Arq Bras Endocrinol Metabol. (2011) 55:54–9. 10.1590/S0004-27302011000100007 21468520

[41] Oswiecimska J Dawidziuk M Gambin T Ziora K Marek M Rzonca S. A Patient with Berardinelli-Seip Syndrome, novel AGPAT2 splicesite mutation and concomitant development of non-diabetic polyneuropathy. J Clin Res Pediatr Endocrinol. (2018) 11:319–26. 10.4274/jcrpe.0227 30563316 PMC6745459

[42] Shetty S Chapla A Kapoor N Thomas N Paul TV A novel variant of the AGPAT2 mutation in generalized congenital lipodystrophy, detected by next generation sequencing. Australas Med J. (2016) 9:164–8. 10.21767/AMJ.2016.2640

[43] Taleban S Carew HT Dichek HL Deeb SS Hollenback D Weigle DS. Energy balance in congenital generalized lipodystrophy type I. Metabolism. (2008) 57:1155–61. 10.1016/j.metabol.2008.04.008 18640396 PMC3259008

[44] Liu Y Li D Ding Y Kang L Jin Y Song J. Further delineation of AGPAT2 and BSCL2 related congenital generalized lipodystrophy in young infants. Eur J Med Genet. (2018) 62:103542. 10.1016/j.ejmg.2018.09.009 30266686

[45] Lupsa BC Sachdev V Lungu AO Rosing DR Gorden P. Cardiomyopathy in congenital and acquired generalized lipodystrophy: a clinical assessment. Medicine. (2010) 89:245–50. 10.1097/MD.0b013e3181e9442f 20616664 PMC3090142

[46] Hussain I Patni N Garg A. Lipodystrophies, dyslipidaemias and atherosclerotic cardiovascular disease. Pathology. (2019) 51:202–12. 10.1016/j.pathol.2018.11.004 30595509 PMC6402807

[47] Lima JG Nobrega LHC Lima NN Dos Santos MCF Silva PHD Baracho MFP Causes of death in patients with Berardinelli-Seip congenital generalized lipodystrophy. PLoS ONE. (2018) 8:e0199052 10.1371/journal.pone.0199052 PMC5993255 29883474

[48] Bhayana S Siu VM Joubert GI Clarson CL Cao H Hegele RA. Cardiomyopathy in congenital complete lipodystrophy. Clin Genet. (2002) 61:283–7. 10.1034/j.1399-0004.2002.610407.x 12030893

[49] Van Maldergem L Magré J Khallouf TE Gedde-Dahl T Jr Delépine M Trygstad O. Genotype-phenotype relationships in Berardinelli-Seip congenital lipodystrophy. J Med Genet. (2002) 39:722–33. 10.1136/jmg.39.10.722 12362029 PMC1734991

[50] Craveiro Sarmento AS Ferreira LC Lima JG de Azevedo Medeiros LB Barbosa Cunha PT Agnez-Lima LF. The worldwide mutational landscape of Berardinelli-Seip congenital lipodystrophy. Mutat Res. (2019) 781:30–52. 10.1016/j.mrrev.2019.03.005 31416577

